# Prepare dispersed CIS nano-scale particles and spray coating CIS absorber layers using nano-scale precursors

**DOI:** 10.1186/1556-276X-9-1

**Published:** 2014-01-01

**Authors:** Jian-Chiun Liou, Chien-Chen Diao, Jing-Jenn Lin, Yen-Lin Chen, Cheng-Fu Yang

**Affiliations:** 1 Electronics and Optoelectronics Research Laboratories, Industrial Technology Research Institute, Hsinchu 31040, Taiwan; 2 Department of Electronic Engineering, Kao Yuan University, Kaohsiung 82151, Taiwan; 3 Department of Applied Materials and Optoelectronic Engineering, National Chi Nan University, Puli 54561, Taiwan; 4 Department of Chemical and Materials Engineering, National University of Kaohsiung, Kaohsiung 82151, Taiwan

**Keywords:** Nano-scale particle, Spray coating method, CIS absorber layer, Annealing

## Abstract

In this study, the Mo-electrode thin films were deposited by a two-stepped process, and the high-purity copper indium selenide-based powder (CuInSe_2_, CIS) was fabricated by hydrothermal process by Nanowin Technology Co. Ltd. From the X-ray pattern of the CIS precursor, the mainly crystalline phase was CIS, and the almost undetectable CuSe phase was observed. Because the CIS powder was aggregated into micro-scale particles and the average particle sizes were approximately 3 to 8 μm, the CIS power was ground into nano-scale particles, then the 6 wt.% CIS particles were dispersed into isopropyl alcohol to get the solution for spray coating method. Then, 0.1 ml CIS solution was sprayed on the 20 mm × 10 mm Mo/glass substrates, and the heat treatment for the nano-scale CIS solution under various parameters was carried out in a selenization furnace. The annealing temperature was set at 550°C, and the annealing time was changed from 5 to 30 min, without extra Se content was added in the furnace. The influences of annealing time on the densification, crystallization, resistivity (*ρ*), hall mobility (*μ*), and carrier concentration of the CIS absorber layers were well investigated in this study.

## Background

In the past, the major developments for the solar cells were on the single-crystalline and multi-crystalline Si-based materials. However, those solar cells will spend too many materials, and they have the shortcoming of the high-temperature-dependence properties, i.e., their efficiencies are critically decreased as the temperature is increased from 40°C to 80°C. Single-crystalline Si-based solar cells, however, have been known to have two major disadvantages of low photoelectric conversion rate and expensive cost of single-crystalline silicon wafer [1]. To overcome those problems, some researchers have examined the II-IV compound semiconductor solar cell [2,3]. Among those, the CuInSe (CIS) and CuIn_1−*x*_Ga_*x*_Se_2_ (CIGS) systems are known to have some advantages such as non-toxicity, long-time stability, and high conversion efficiency [4]. For that, the CIS and CIGS thin films are being studied as promising absorber material for high-efficiency, low-cost, thin-film solar cells. The inherent advantages of the direct band gap material CIS and CIGS thin-film solar cells are based on its high absorption and therewith low layer thickness required for light absorption. The resultant potential for cost reduction, light weight, and flexible applications makes the CIS and CIGS absorber layer an all-round candidate for cheap large-area module technology as well as special architectural and space applications [5].

To further increase the applicability and profitability, a further improvement in the fabrication process of the CIS and CIGS thin films is necessary. In the past, CIS and CIGS absorber layers could be prepared by various methods, sputtering and co-evaporation are two of the most popular methods to deposit CIS and CIGS absorber layers. Wuerz et al. used the co-evaporation process to fabricate the highly efficient CIS absorber layers on different substrates [5] and Hsu et al. used the sputtering and selenization processes to deposit the CIGS absorber layers [6]. Traditional vacuum methods are too complicated and difficult because those methods require a large number of expensive equipments, when the number of process parameters increases. Also, there are many non-vacuum methods were investigated, including spray pyrolysis [7], electrodeposit [8], and non-vacuum particle-based techniques [9]. It can be easily assumed that the process cost could be lowered by non-vacuum thick-film process such as screen printing, though nano-sized powders of the CIS and CIGS precursors are needed for the paste. For synthesis of the nano-sized CIS and CIGS powders, the solvothermal method has been mainly adopted, for it can easily control particle characteristics and produces much amount of powder [10]. However, single-phase powders of CIS and CIGS have never been synthesized by the solvothermal method [11-13].

The spray pyrolysis method (SPM) is a very important non-vacuum deposition method to fabricate thin films because it is a relatively simple and inexpensive non-vacuum deposition method for large-area coating [14]. In this study, the micro-sized CIS powder was synthesized by the hydrothermal process by Nanowin Technology Co. Ltd. Because the formed CIS powder was aggregated in the micro-scale, for that we ground the CIS powder by the ball milling method. Particle-size change during process has been observed by Field-emission scanning electron microscopy (FESEM) and X-ray diffraction (XRD) patterns to examine the effect of adding dispersant or not and grinding time on particle size. A SPM method was used to develop the CIS absorber layers with high densification structure. However, only few efforts had been made to systematically investigate the effects of thermal-treated parameters in a selenization furnace on the physical and electrical properties of the CIS absorber layers. We would investigate the effects of annealing parameters on the physical and electrical properties of the CIS absorber layers. The feasibility of the crystalline phase CIS by controlling RTA-treated temperature and time has been checked.

## Methods

**Figure 1:**
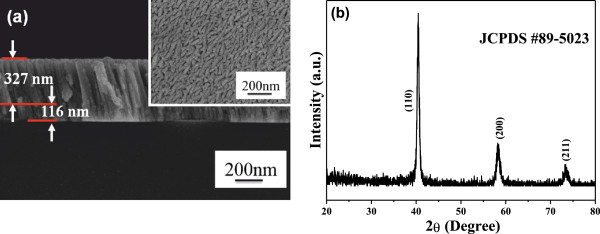
Cross section and surface morphologies (in the upset) (a) and XRD pattern of the deposited bi-layer Mo electrode (b).

In the past, several materials have been with the subjects of experiment for use as a back contact electrode for CIS and CIGS thin films, such as W, Ta, Nb, Cr, V, or Ti. Molybdenum (Mo) thin films are widely used as a back contact electrode for CIS- and CIGS-based solar cells, because of its inertness and high conductivity [15]. The back electrode layer functions as a barrier that hinders the diffusion of impurities from the substrates into the absorber layers. In this study, the corning eagle XG glass (thickness was 0.7 mm) with the size 20 mm × 10 mm was used as substrates to deposit the bi-layer-structured Mo electrode at room temperature in pure argon. After the surfaces of the glass substrates were cleaned, then they put into the sputter. At first, the chamber was pumped to 8 × 10^−6^ Torr, the first layer of Mo was deposited at the deposition parameters of power of 50 W, working pressure of 10 m Torr, and Ar flow rate of 70 sccm for 6.5 min; the second layer of Mo was deposited at the deposition parameters of power of 50 W, working pressure of 5 m Torr, and Ar flow rate of 20 sccm for 29 min, respectively. The first layer had a thickness of approximately 116 nm and the second layer had a thickness of approximately 327 nm, as Figure 1a shows. The surface of the deposited Mo electrode was shown in the inset of Figure 1a, the bar-typed grains with length of 40 to 160 nm and width of 20 to 32 nm were obtained. The X-ray diffraction pattern was used to measure the crystallization of the bi-layer-structured Mo electrode, the diffraction peaks of (110), (200), and (211) were apparently observed. The diffraction peaks matched the 2*θ* pointed by JCPDS #89-5023 for Mo metal. The high-purity copper indium selenide-based powder (CIS) was synthesized and formed by hydrothermal process by Nanowin Technology Co. Ltd. Because the CIS precursor was aggregated into micro-scale particles, the milling ball with the average diameter of 0.2 mm was used to grind them from 1 to 4 h. With and without addition of 1 wt.% dispersant (KD1) was also used as the parameter to compare the grinding effect. The morphologies of those ground CIS powders were observed using field-emission scanning electron microscope, and their crystalline structures were measured using X-ray diffraction patterns with Cu Kα radiation (*λ* = 1.5418 Å).

After finding the optimum grinding time and KD1 content, the 6 wt.% CIS particle was dispersed into isopropyl alcohol (IPA) to get the solution for SPM to prepare the CIS absorber layers. The organic/CIS composite films were formed by spray coating method (SCM) on Mo/glass, and then the organic/CIS composite films were annealed for 5 min by the rapid temperature annealing (RTA) process in selenization furnace (the chamber size is 5 cm × 5 cm × 4 cm) under different annealing parameters to remove the used organic and crystallize the CIS absorber layers. Then, 550°C was used as the annealing temperature, without extra Se content was put in the furnace during the annealing process, and the annealing time was changed from 5 to 30 min. After annealing process, the crystalline structure was examined using the XRD pattern and the surface morphology and cross section observations of the CIS absorber layers were examined by FESEM, respectively. The electrical resistivity and the Hall-effect coefficients were measured using a Bio-Rad Hall set-up.

## Results and discussion

**Figure 2:**
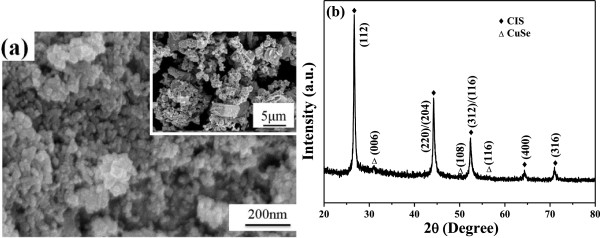
CIGS precursors observed in (a) nano-scale (nm) and micro-scale (μm, in the upset) morphologies (b) XRD pattern.

The surface morphology and microstructure of the CIS precursor are investigated using the FESEM observations and the results are shown in Figure 2. As Figure 2a shows, the CIS precursor obtained by the hydrothermal process was really in the nano-scale (nm). As shown in the inset of Figure 2a, even the original CIS particles were in the nano-scale, the micro-scale (μm)-aggregated structure was apparently observed. The XRD pattern of the CIS precursor was investigated and the result is shown in Figure 2b. As shown in Figure 2b, the mainly crystalline phase was CIS, and the almost undetectable secondary CuSe phase was observed. For the further application of the CIS powder in the printing method, the CIS should be ground into the nano-scale particles.

**Figure 3:**
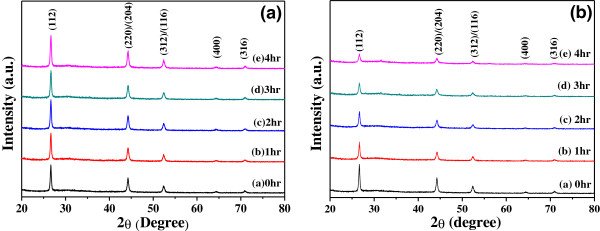
**XRD patterns of the CIS precursors grinding using a 2-mm ZrO**_**2 **_**ball (a) without KD1 dispersant and (b) with KD1 dispersant.**

The XRD patterns of the CIS precursor were investigated under various grinding time and with and without 1 wt.% KD1, and the results are shown in Figure 3. As shown in Figure 3, only the diffraction peaks of the CIS phase were observed in the ground powders. The 2*θ* values of the diffraction peak for the CIS particle under differently treated process had no apparent shift. This result suggests that the crystalline phases of the CIS particle are not changed as the grinding process is used. For the ground CIS precursor without KD1 addition, the full width at half maximum (FWHM) value of the (112) peak was 0.37°, 0.37°, 0.38°, 0.38°, and 0.38° as grinding time was 1, 2, 3, and 4 h, respectively, as Figure 3a shows; as shown in Figure 3b for ground CIS precursor with KD1 addition, the FWHM value of the (112) peak was 0.38°, 0.43°, 0.47°, and 0.52°, as grinding time was 1, 2, 3, and 4 h, respectively. The increase in the FWHM values of the (112) peak suggests that the particle sizes of the CIS powder decrease with increasing grinding time. However, the variations in the particle sizes of the ground CIS powders are dependent on the KD1 concentration and grinding time and they are not easily calculated from the surface observation. In the past, the particle size can be estimated using the Scherrer’s formula [16]:

(1) $$D=\frac{\mathrm{k\lambda}}{B\cos\theta },$$

where *λ* is the X-ray wavelength, *B* is the full width of height maximum of a diffraction peak, *θ* is the diffraction angle, and *k* is the Scherrer’s constant of the order unity for usual crystal. For CIS powder ground without KD1 addition it aggregated into micro-scale particles with the diameter in the range of 1.3 to 6 μm (not shown here). However, as the KD1 was added, the CIS powder was ground into nano-scale after 4 h, and it had the average particle sizes approximately 20 to 50 nm (also not shown here). Those results indicate that as KD1 is added as dispersant, the particle sizes of the CIS power are really decreased from micro-scale to nano-scale.

**Figure 4:**
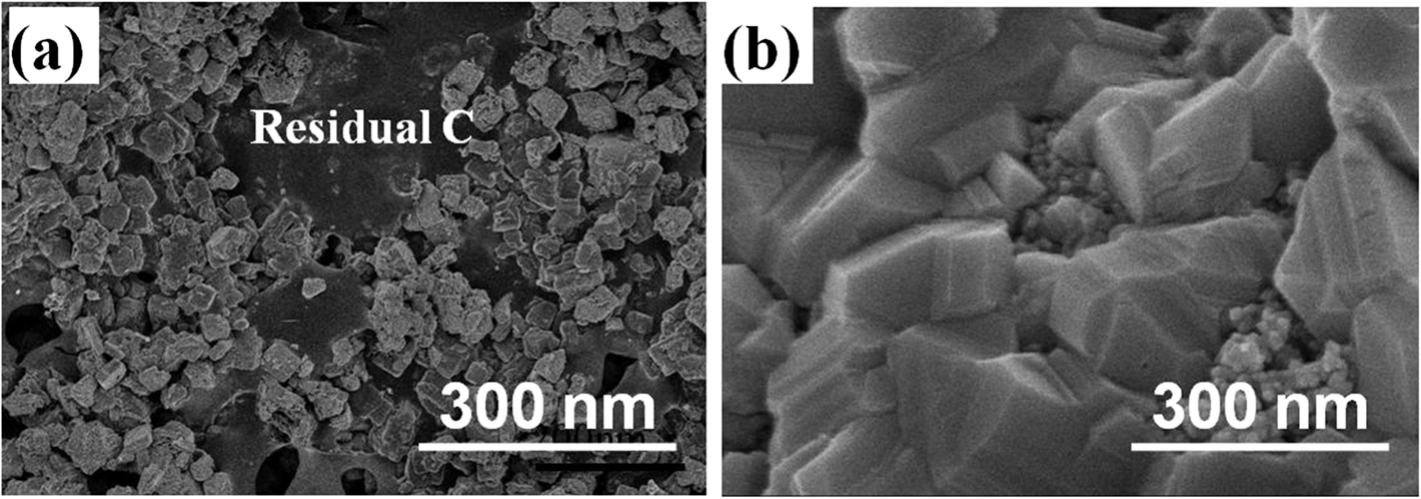
Surface morphologies of the RTA-treated CIS absorber layers on the Mo/Glass substrates (a) at 450°C and (b) at 500°C for 10 min.

Figure 4 shows the surface morphology of the CIS absorber layers on the Mo/Glass substrates, RTA was carried out at different temperatures for 10 min in a selenization furnace and without extra Se addition. As Figure 4 shows, residual carbon was really observed in the 450°C-annealed CIS absorber layer (Figure 4a), and the carbon was not observable in the 500°C-annealed CIS absorber layer (Figure 4b). From the analyses of energy-dispersive spectrometers (EDS) carbon cannot be undetected in the CIS absorber layers (not shown here). Those results suggest that the as the CIS absorber layers are printed on the Mo/glass substrates, 500°C is enough for crystallized CIS and eliminated the dispersant KD1. For that, the CIS absorber layer was annealed at 550°C at different time, without extra Se was added into selenization furnace.

**Figure 5:**
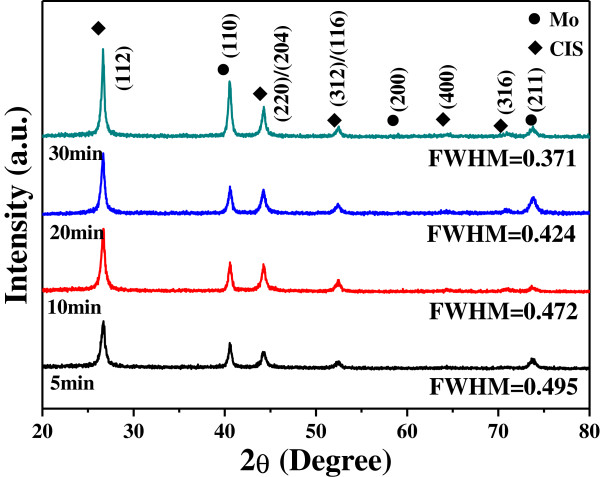
XRD patterns of the CIS absorber layer annealed at 550°C as a function of annealing time.

The XRD patterns of the CIS absorber layers as a function of annealing time were investigated, the annealing time was set at 550°C and the results are shown in Figure 5. The mainly crystalline peak of the CIS absorber layers was the (112) and the secondary CuSe phase was not observed. Even annealing time was increased from 5 to 30 min, the all (112) peaks revealed in Figure 5 were situated at 2*θ* around 26.66°. This result suggests that annealed at 550°C and as annealing time was changed from 5 to 30 min, the lattice constant and the composition of the CIS absorber layers have no apparent change. As the CIS absorber layers are used to fabricate the thin film solar cells, the formation of secondary phases will degenerate the efficiency. Figure 5 also shows that the no secondary phases were observed in the annealed CIS absorber layers, even 30 min was used as annealing time. This result suggests that 550°C is a suitable annealing temperature for the CIS absorber layers because no secondary phases are formed. The full width at half maximum (FWHM) value of the (112) peak of the CIS absorber layers was 0.496, 0.472, 0.424, and 0.371 as the annealing time was 5, 10, 20, and 30 min, respectively. Also, the relative diffraction intensity of (112) peak had no apparent change as the annealing time increased from 5 to 30 min, as indicated by the XRD patterns shown in Figure 5. Longer annealing time resulting in better crystalline structure is the reason to cause this result. This is because as longer time is used to anneal the CIS absorber layers, the number of thin film defects decreases and the crystallization of the CIS absorber layer is improved, then the FWHM value decreases.

**Figure 6:**
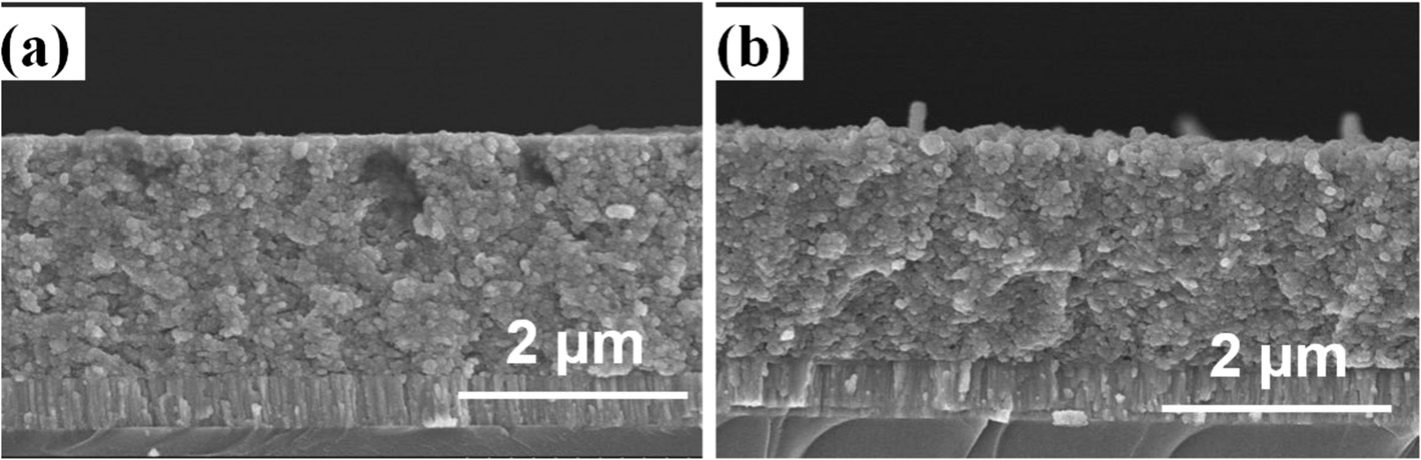
Cross section observations of the CIS absorber layers as a function of annealing time (a) 5 min and (b) 20 min.

The cross section observations of the CIS absorber layers as a function of annealing time are shown in Figure 6, the annealing time for Figure 6a,b was 5 and 20 min, respectively. As Figure 6a,b show, the thicknesses of the annealed CIS absorption layers were around 1,905 ± 53 nm. This result proves that we can deposit the CIS absorption layers with uniform thickness by the spray coating method. The cross section morphologies also show that the densified structures were really obtained in the 5- and 20-min-annealed CIS absorption layers.

**Figure 7:**
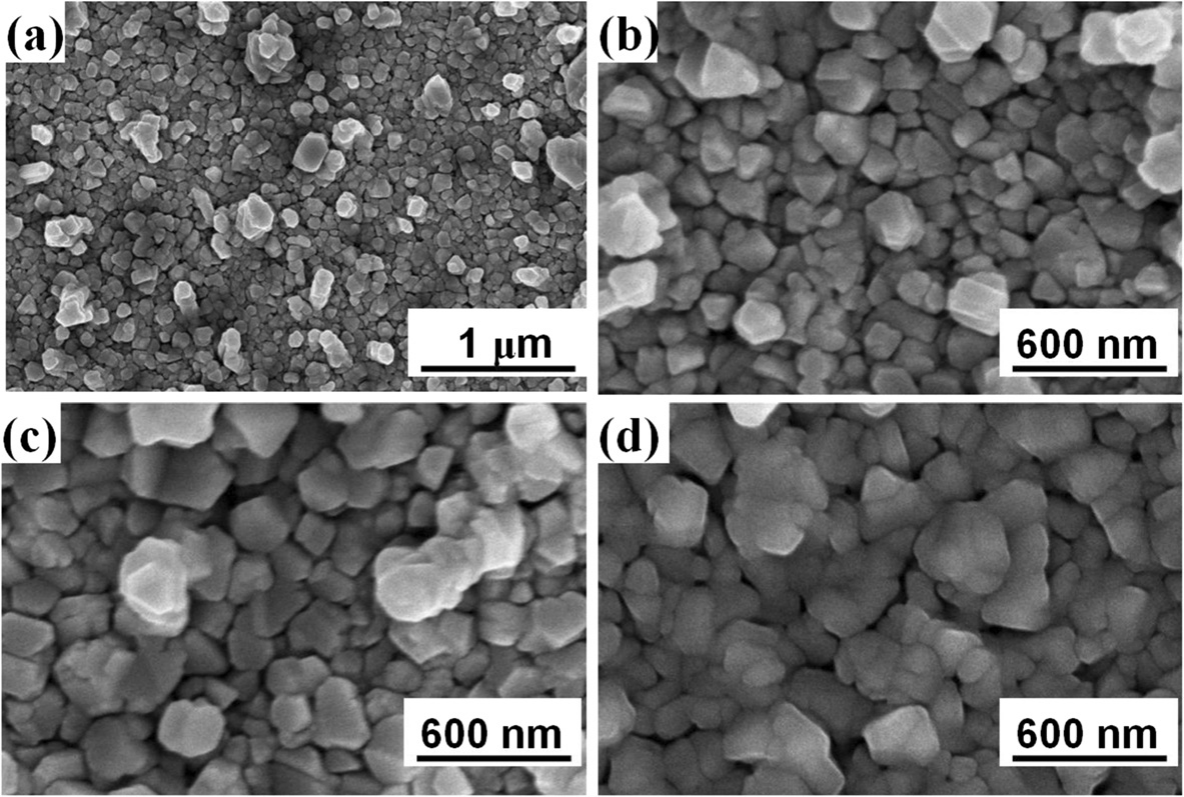
Surface morphologies of the CIS absorber layers as a function of annealing time (a) 5, (b) 10, (c) 20, and (d) 30 min, respectively.

The surface morphologies of the CIS absorber layers under different annealing time are shown in Figure 7, which indicates that the annealing time has a significant effect on the CIS absorber layers’ surface morphologies. As Figure 7 shows, annealing at 55°C, all CIS thin films had a densified structure. Those results prove that 550°C is high enough to improve the densification and grain growth of the CIS absorber layers, and a roughness surface is obtained. When the annealing time was increased from 5 to 30 min, the roughness and grain sizes were apparently increased and only nano-scale grains were observed. The increase in the grain sizes is caused by the increase in the crystallization of the CIS absorber layers, the decrease in the FWHM values proves this result.

**Figure 8:**
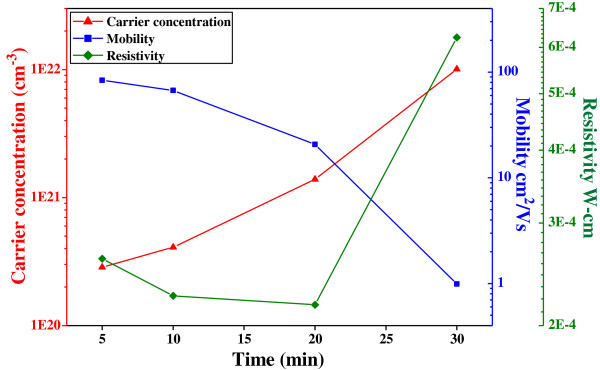
**Resistivity ( *ρ *), hall mobility ( *μ *), and carrier concentration ( *n *) of the CIS absorber layers, annealed at 550°C**.

Figure 8 shows variations in the electrical properties of the CIS absorber layers annealed at 550°C as a function of annealing time. When the CIS absorber layers are deposited on a glass substrate by SCM and annealing process, many defects result and inhibit electron movement. As the various annealing time is used, two factors are believed to cause an increase in the carrier mobility of the CIS absorber layers. First, the longer annealing time enhances the densification and crystallization, which will decrease the numbers of defects and pores in the CIS absorber layers and will cause the decrease in the inhibiting of the barriers electron transportation [17]. Second, as the annealing time is too long, the secondary phase of the CIS absorber layers will appear because of the vaporization of Se. In this study, the carrier concentration increased with increasing annealing time and reached a maximum of 1.01 × 10^22^ cm^–3^ at 30 min. Thus, the mobility decreased with increasing annealing time and reached a minimum of 1.01 cm^2^/V-s at 30 min. The resistivity of the CIS absorber layers is proportional to the reciprocal of the product of carrier concentration *N* and mobility *μ*:

(2) p=1/Neμ.

Both the carrier concentration and the carrier mobility contribute to the conductivity. The resistivity of the all CIS absorber layers were in the region of 3.17 to 6.42 × 10^−4^ Ω-cm and the minimum resistivity of 2.17 × 10^−4^ Ω-cm appeared at the 20 min-annealed CIS films.

## Conclusions

After finding the optimum grinding time, the CIGS powder had the average particle sizes approximately 20 to 50 nm. As the grinding time was 1, 2, 3, and 4 h, the FWHM values of the (112) peak were 0.37°, 0.37°, 0.38°, 0.38°, and 0.38° for CIS without KD1 addition and the FWHM values of the (112) peak were 0.38°, 0.43°, 0.47°, and 0.52° for CIS with KD1 addition, respectively. As annealing temperature was 550°C and annealing time of CIS absorber layers was 5, 10, 20, and 30 min, the FWHM values of the (112) peak was 0.496, 0.472, 0.424, and 0.371, respectively. In this study, the thicknesses of the annealed CIS absorption layers were around 1,905 ± 53 nm. The carrier concentration had a maximum of 1.01 × 10^22^ cm^–3^ at 30 min and the mobility had a minimum of 1.01 cm^2^/V-s at 30 min. The resistivity of all the CIS absorber layers was in the region of 3.17 to 6.42 × 10^−4^ Ω-cm and the minimum resistivity of 2.17 × 10^−4^ Ω-cm appeared at the 20-min-annealed CIS films.

## Competing interests

The authors declare that they have no competing interests.

## Authors’ contributions

JCL proposed an idea to fabricate the CIS absorber layers and helped in the Mo deposition. CCD, JJL, and YLC participated in the experimental process and helped in the data analysis. CFY also proposed an idea to fabricate the CIS absorber layers and wrote the paper. All authors read and approved the final manuscript.
